# Re-thinking the inclusion of race in British hypertension guidance

**DOI:** 10.1038/s41371-021-00601-9

**Published:** 2021-09-10

**Authors:** Dipesh P. Gopal, Grace N. Okoli, Mala Rao

**Affiliations:** 1 Centre for Primary Care, Wolfson Institute of Population Health, Barts and The London School of Medicine and Dentistry, Yvonne Carter Building, London, UK; 2 Ethnicity and Health Unit, Department of Primary Care and Public Health, Imperial College London, London, UK

**Keywords:** Health care, Renovascular hypertension

Last year saw the resurgence of the Black Lives Matter movement and a renewed global call for racial justice. The disproportionate impact of the covid-19 pandemic added further weight to a clear moral argument for equality within wider society. Against that background, in medicine too, long held biological assumptions about the influence of race in the diagnosis and management of illness are being evaluated, in light of expanding scientific knowledge.

Traditionally medical research typically relies on the concept of finding a single clear truth or ‘scientific’ answer. In this way researchers are seen as objective observers who have an ability to control for research biases. This is the premise of quantative research and how race is currently seen as a proxy for understanding ethnic differences in health. However, research data and clinical guidance do not develop in a vacuum and are shaped by society and the politics of their time [1]. An alternative view to traditional medical research is that scientists are not exempt from the influence of bias, and it is impossible to correct for our biases in research. This is the premise of qualitative research. Self-ascribed race is used to provide insights into the rates of hypertension or blood pressure responses to medication without appreciating the context in which they occur. These opposing ideologies challenge the very core of using race, a poorly defined social construct, within medical research [2].

## Current guidance on the treatment of hypertension

The current British National Institute for Health and Care Excellence guidelines [3] recommend that people who do not have Black African or Black Caribbean heritage are offered ACE inhibitors initially, in preference to calcium channel blockers or thiazide diuretics. In contrast, people who have Black African or Black Caribbean heritage are offered calcium channel blockers initially but not offered angiotensin-converting-enzyme (ACE) inhibitors. This is because all people with Black heritage are thought to be low renin responders so therefore less responsive to ACE inhibitors. However, ACE inhibitors may be offered later if calcium channel blockers are insufficient to maintain adequate blood pressure control. The inconsistency in this recommendation as well as many others was highlighted in a previous publication in this journal [4]. This correspondence was sent to the British NICE hypertension committee who considered the presented evidence but deferred guidance change (personal correspondence) until the next review, the date for this being uncertain at the current time.

The British NICE guidelines are also notable in their contrast to US [5], South African [6] and International [7] hypertension guidance, as none of these include race as a determinant of treatment decisions.

This perspective article summarises the evidence base for the guidance on the treatment of hypertension published by NICE and explains why the inclusion of race as a criterion for treatment is questionable and deserves to be reviewed as a matter of urgency.

## What do we know about blood pressure and ethnicity?

First, there is a minimal difference in blood pressure response to ACE inhibitors when comparing Black and White people. A 2013 meta-analysis using results from 13 trials found that there was 4.6 mmHg lower blood pressure response to ACE inhibitors for 2505 Black people compared to 10427 White people [8]. Twelve trials were based in the USA with the remaining trial based in the Netherlands. Whilst there was low risk of publication bias, we wonder whether this data could reliably apply to UK populations. None of the trials considered known confounders such as socioeconomic inequalities [9] or perceived discrimination [10]. A 2020 UK cohort study, using data from 2007 and 2017, found that 4010 Black people had 5.6 mmHg lower anti-hypertensive effect to ACE inhibitors and angiotensin receptor blockers (ARBs) compared to 150704 non-Black (including people with Asian heritage) at 12 weeks [11]. This effect persisted one year later without controlling for confounders. Despite this, the overlapping confidence intervals in changes in blood pressure indicates that Black people had a non-significant blood pressure lowering effect to ACE inhibitors and ARBs compared to non-Black people in the UK context. This is more representative of the UK population, compared to the 2013 meta-analysis [8] using populations from the USA and the Netherlands.

Second, the inclusion of race within the hypertension guidelines assumes a distinct biological and genetic homogeneity amongst all Black and White people which contradicts contemporary thought [12]. The blood pressure lowering effect of ACE inhibitors is thought to be smaller in Black people as outlined above. Some report that this is due to inherent biological differences between Black and non-Black people such as a lower renin response [13]. Despite this, renin activity varies independently of blood pressure casting doubt on this theory [14, 15]. Genetic differences between members of different races are much smaller than differences between members of the same race [16, 17]. Furthermore, race is a social construct, not a biological one [12]. The classification of Black African or Caribbean lineage is self-ascribed and does not account for people with mixed heritage or people with distant Black ancestry such as grandparents or great-grandparents. People who describe themselves as white may very well have distant Black ancestry. Consequently, it becomes much more difficult for clinicians to base treatment decisions on race and who is judged to be black, especially given immigration from Asia and Africa to the UK over the last 70 years [18].

Third, the NICE UK hypertension guidelines are inconsistent, given that initial treatment is assigned on race, but subsequent second line management is not. If race is a criterion for treatment, why would it become irrelevant once initial treatment is ineffective?.

Fourthly, there is no mention of race-based guidance for the use of ACE inhibitors in British NICE guidance such as in heart failure [19] or post-myocardial infarction [20] possibly as the cardio-protective rather than anti-hypertensive effect of prescribed medications is desired.

A factor that we must highlight is the reported increased risk of ACE inhibitor angioedema within Black people that is described in the NICE guidance evidence reviews [21]. A 2006 systematic review [22] compared the risk of ACE-inhibitor angioedema between 55252 Black and 133964 non-Black patients. The data was from 4 US cohort and randomised control trials and 1 multinational trial using US, European, Israeli, Russian and Australian populations. The incidence of ACE-inhibitor angioedema for Black people was 0.43% compared to 0.18% for non-Black people. This would give an almost 3 times higher odds ratio of angioedema for Black people, but the absolute risk is small. A 2005 US trial [23] that was not included in the meta-analysis found a similar odds ratio but higher incidence of angioedema at 1.62% in 1237 Black people compared to 11397 non-Black people. This trial was cited as evidence to use ARBs over ACE inhibitors for Black people as part of recent international hypertension guidance [7]. Interestingly it is not clear how race is defined and whether that was defined by participants or researchers. Both the meta-analysis and trial contain predominantly US data which once again may have limited applicability to British guidelines let alone international guidance. In addition, ACE-inhibitor associated angioedema is apparently not relevant in the treatment of heart failure, reinforcing the view that race as a criterion is unjustified.

Another consideration is cardiovascular outcomes which have been flagged from a recent meta-analysis [24] comparing the effect of ACE inhibitors or ARBs against other antihypertensives such as calcium channel blockers amongst Black people with hypertension with 4-to-6-year follow-up. The four US studies included in this analysis, involved 38,983 participants but since all studies did not involve all types of antihypertensives, it was not possible to include all study data in subgroup analysis. There were no significant differences in all-cause mortality or any cardiovascular outcome when comparing ACE inhibitors/ARBs to beta-blockers. However, ACE inhibitors/ARBs had 56 and 59% significantly higher risk of stroke compared to calcium channel blockers and diuretics respectively. For example, the absolute risk of stroke for Black people who were taking calcium channel blockers was 2.57% compared to 3.99% for those taking ACE inhibitors/ARBs. Once again, US data may not be applicable to the UK given differing regional and national socioeconomic inequalities [9] and perceived stress [25]. The meta-analysis concluded that the lack of measurement of blood pressure reduction in response to the analysed anti-hypertensives makes it hard to speculate on the cause of the higher stroke rate. Furthermore, not all the studies used multivariate regression models to adjust for known confounders such as socioeconomic inequalities. The meta-analysis compounds results for ACE inhibitors and ARBs making it hard to determine causation based on positive correlation between medication and stroke risk.

## Conclusion

There is an urgent need to re-consider the inconsistencies in using ACE inhibitors to lower blood pressure for Black people and the rationale of assigning antihypertensives based on self-assigned race. Furthermore, there are inconsistencies in the inclusion of race in all guidelines and the reliance on US data without context for identifying short-term and long-term effects of ACE inhibitors such as angioedema or risk of stroke. There is a real concern that poorly defined self-assigned race without context, would encourage a historic perspective of racial pathology that lacks a robust scientific evidence-base to persist, and to allow persistence of existing health inequalities [26]. A more nuanced approach [27] that incorporates the fields of genetics, physiology, pharmacokinetics and social science would be more applicable to current scientific research approaches, as it would use mixed methods to integrate the complex intersection of race, ethnicity, society and medicine in the antihypertensive prescribing guidance, and indeed in all treatment guidelines in the future.
